# Associations between academic burnout, resilience and life satisfaction among medical students: a three-wave longitudinal study

**DOI:** 10.1186/s12909-022-03326-6

**Published:** 2022-04-05

**Authors:** Qinghua Wang, Wei Sun, Huazhang Wu

**Affiliations:** 1 Institute of Foreign Languages, China Medical University, No. 77 Puhe Road, Shenyang North New Area, Shenyang, Liaoning Province People’s Republic of China; 2 Research Center for Universal Health, School of Public Health, China Medical University, No. 77 Puhe Road, Shenyang North New Area, Shenyang, Liaoning Province People’s Republic of China; 3 Department of Health Service Administration, College of Health Management, China Medical University, No. 77 Puhe Road, Shenyang North New Area, Shenyang, Liaoning Province People’s Republic of China

**Keywords:** Medical students, Academic burnout, Resilience, Life satisfaction, Longitudinal study, Temporal relationships, Mediation

## Abstract

**Background:**

Research shows that there are connections among academic burnout, resilience, and life satisfaction in medical students. However, no study has yet examined the temporal relationships between academic burnout, resilience, and life satisfaction among medical students. This longitudinal study aimed to examine the temporal associations between academic burnout, resilience, and life satisfaction and to explore the possible mediating role of resilience in the relationship between academic burnout and life satisfaction among medical students.

**Methods:**

This is a three-wave longitudinal study covering the preclinical education period of 20 months. From October 2018 to June 2020, a total of 190 students majoring in clinical medicine filled out the Chinese College Student Academic Burnout Inventory (CCSABI), the Connor-Davidson Resilience Scale (CD-RISC) and the Satisfaction With Life Scale (SWLS) three times. Cross-lagged models were constructed to examine the temporal relationships between academic burnout, resilience, and life satisfaction and longitudinal mediation models were constructed to explore the possible mediating role of resilience in the association of academic burnout with life satisfaction.

**Results:**

Among medical students, resilience uni-directionally and positively predicted life satisfaction, while academic burnout uni-directionally and negatively predicted life satisfaction. However, the temporal association between resilience and academic burnout was negative and somewhat bidirectional. Resilience had a significant mediating effect on the relationship between academic burnout and life satisfaction in medical students.

**Conclusions:**

Medical educators need to identify and take effective measures to combat academic burnout problems which can lead to reduced life satisfaction among medical students. Resilience-based interventions may be promising in buffering the negative impacts of academic burnout and improving life satisfaction. It is recommended that effective resilience-promotion interventions be developed and implemented in medical education to help enhance medical students’ psychological well-being.

**Supplementary Information:**

The online version contains supplementary material available at 10.1186/s12909-022-03326-6.

## Background

Medical education is a demanding process full of difficulties and challenges, and different stressors medical students encounter in the process of learning medicine may lead to mental health problems such as academic burnout [1–3]. The concept of “burnout” was initially raised in the occupational field and according to Maslach, burnout is a syndrome characterized by feeling emotionally exhausted, depersonalized, and a low sense of achievement at work [4]. A large number of early studies focused on occupational burnout and it was found that burnout problems are prevalent among health professionals [5–9]. Recently, however, more research has been conducted on academic burnout in the learning environment and academic burnout problems among medical students have aroused great concern worldwide [1–3, 10–12].

Academic burnout, also known as school burnout or learning burnout, is a kind of burnout syndrome students acquire in the academic environment. Lian et al. defined academic burnout as a state of prolonged negative emotions towards study, inappropriate learning behaviors and reduced sense of academic accomplishment [13]. Students with academic burnout syndrome were shown to have decreased interest and motivation in study, but increased levels of negative emotions such as anxiety, depression, fatigue and frustration [14, 15]. Research shows that demographic factors (e.g. age, gender, grade), contextual factors (e.g. learning environment, intensive curriculum), social factors (e.g. social support, interpersonal relationships), and psychological factors (e.g. psychological resources, mental health) are all associated with burnout syndrome among medical students [10, 16]. Our study focused on psychological factors which are found to be closely related to academic burnout in medical students [2]. Psychological resources are important psychological factors that influence a person’s perceived control over the stressful environment [17]. A student who lacks psychological resources may feel overwhelmed by heavy academic workload or have a prolonged sense of failure as a result of ineffective pressure management [18].

An important psychological resource researchers paid much attention to in medical education is resilience. Connor and Davidson defined resilience as a personal quality which enables individuals to thrive when facing adversities [19]. Individuals with high resilience levels were found to be persevering, self-reliant, equanimous and they had clear life goals for which they persistently strove to achieve despite challenges or difficulties [20]. Thus, resilience, as a personal psychological resource, is essential for one to cope with adversity effectively and to persevere in reaching one’s goals under stress [21]. Although some cross-sectional studies show that resilience was negatively related to academic burnout among medical students [22, 23], we found that no studies have explored the temporal association of resilience with academic burnout. Therefore, the first aim of our study was to examine the temporal relationship between resilience and academic burnout among medical students by using a three-wave cross-lagged model.

Research shows that academic burnout among medical students exerted a considerable impact on their psychological health [22, 23]. A two-wave longitudinal study by Ríos-Risquez and colleagues found that emotional exhaustion, a dimension of Maslach Burnout Inventory Student Survey (MBI-SS), was the strongest negative predictor of nursing students’ psychological well-being [24]. Life satisfaction, as a core component of subjective well-being and a key indicator of psychological health, was defined as personal judgments of overall life situation based on one’s self-set standards and one’s own expectations [25]. Although correlational studies have demonstrated the negative association of burnout with life satisfaction among medical students [26, 27], to the best of our knowledge, no research has yet explored the temporal relationship between academic burnout and life satisfaction, so the second aim of the current study was to examine this relationship among medical students by using a three-wave cross-lagged model.

The protective role of resilience for medical students’ mental health has been demonstrated in a large number of studies [23, 24, 28–38]. Although the positive correlation of resilience with life satisfaction among medical students has been confirmed in some cross-sectional studies [39, 40], we did not find any research examining the temporal association between resilience and life satisfaction. Thus, as the third aim of the present study, we tried to explore the temporal relationship between resilience and life satisfaction among medical students by using a three-wave cross-lagged model.

Finally, in view of the above-mentioned studies on the close associations between academic burnout, resilience and life satisfaction among medical students, and since previous research has revealed that resilience is an important psychological resource and an essential mental health protector, we hypothesized that resilience may play a mediating role in the relationship between academic burnout and life satisfaction among medical students. Thus, the fourth aim of the present study was to test this hypothesis by using longitudinal mediation models.

To summarize, the current research aimed to examine the temporal relationships (i) between resilience and academic burnout; (ii) between academic burnout and life satisfaction; (iii) between resilience and life satisfaction and (iv) to explore the possible mediating role of resilience in the relationship between academic burnout and life satisfaction among medical students by adopting a three-wave longitudinal design.

## Methods

### Study design and subjects

This three-wave longitudinal study was conducted in China Medical University, a key medical university in Northeast China. In China Medical University, undergraduate medical education for students majoring in clinical medicine takes 5 years, and there are about 20 classes of students in this major in each grade with approximately 30 students in each class. In October 2018, we randomly selected eight classes of first-year undergraduate students whose major is clinical medicine to participate in our survey. From the 253 medical students recruited, 212 students agreed to participate (response rate: 83.79%) in Wave 1 (88 males and 124 females; *M*_age_ = 18.09 years, *SD*_age_ = 0.66 years, T_1_). In October 2019, in the recruitment for subjects for Wave 2, 222 medical students agreed to participate (response rate: 87.75%; 94 males and 128 females, *M*_age_ = 19.10 years, *SD*_age_ = 0.65 years, T_2_). As for Wave 3, 216 medical students participated in June 2020 (response rate: 85.38%; 94 males and 122 females, *M*_age_ = 19.74 years, *SD*_age_ = 0.78 years, T_3_). Among the subjects, 190 medical students (81 males and 109 females) completed all three waves of the survey and we used the data from this group of 190 medical students for the cross-lagged analyses in our study (for the homogeneity tests of 190 medical students as the representative for 212, 222 and 216 subjects respectively in the three waves, please refer to the Additional file 1).

### Ethical approval

An online survey tool called Wenjuan Xing was used to distribute questionnaires and trained research investigators explained the purpose of the study to medical students. Participation was voluntary and students were assured that the collected data would be kept confidential and would be used for research purposes only. Students were told that they could withdraw from the survey anytime if they wanted to without any punishment. This study was approved by the Institutional Review Board of China Medical University and was conducted according to the Declaration of Helsinki (59th WMA General Assembly, 2008). Medical students who agreed to participate completed the questionnaires and the informed consent forms online.

### Measures

#### Academic burnout

Medical students’ academic burnout levels were measured by the Chinese College Student Academic Burnout Inventory (CCSABI) [13]. With reference to Maslach Burnout Inventory, Lian and colleagues developed the 20-item CCSABI considering Chinese college students’ psychological characteristics. CCSABI included three subscales: Low Mood (8 items), Improper Behavior (6 items) and Low Achievement (6 items). Example items in CCSABI are “I find it difficult to maintain enthusiasm in study.” and “Until now, I have already exploited my full potential in college study.” Each item was scored on a 5-point Likert Scale ranging from 1 “not true of me at all” to 5 “very true of me” and after the negatively worded items were reverse scored, the sum score was calculated with a higher sum score indicating a higher level of academic burnout. CCSABI has been demonstrated to be a reliable measurement tool among Chinese medical students in previous research [18, 41]. In the current study, Cronbach’s alpha coefficients for CCSABI were 0.872 (T_1_), 0.907 (T_2_) and 0.890 (T_3_), respectively.

#### Resilience

Medical students’ resilience levels were assessed by the 25-item Connor-Davidson Resilience Scale (CD-RISC) [19]. Students rated on the 5-point Likert scale based on their own judgments about how well the item described them from 1 “not true of me at all” to 5 “true of me nearly all the time”. Example items in CD-RISC are “I can deal with whatever comes.” and “Even though there is little hope for something, I would not easily give up.” The total score was calculated and a higher total score indicated a higher level of resilience. The Chinese version of CD-RISC demonstrated sound psychometric properties among medical students in previous research [39, 40]. In the present study, Cronbach’s alpha coefficients for CD-RISC were 0.945 (T_1_), 0.947 (T_2_) and 0.966 (T_3_), respectively.

#### Life satisfaction

The 5-item Satisfaction With Life Scale (SWLS) developed by Diener et al. [25] was used to measure medical students’ overall life satisfaction levels. Each item was scored on a 7-point Likert scale from 1 “strongly disagree” to 7 “strongly agree” and then scores were summed up with a higher total score signifying a higher level of global life satisfaction in medical students. Example items in SWLS are “In most ways, my life is close to my ideal.” and “If I could live my life over, I would change almost nothing.” The Chinese version of SWLS has demonstrated sufficient reliability among medical students [26, 39–41]. In the current research, Cronbach’s alpha coefficients for SWLS were 0.879 (T_1_), 0.913 (T_2_) and 0.909 (T_3_), respectively.

### Data analysis

Means, standard deviations and Pearson correlation coefficients of academic burnout, resilience and life satisfaction were calculated using SPSS version 22.0. Three cross-lagged path models were constructed and examined by AMOS version 24.0. For model fit, six indices were adopted, comprising Chi-square divided by degrees of freedom (χ^2^/df), Goodness of Fit Index (GFI), Adjusted Goodness of Fit Index (AGFI), Comparative Fit Index (CFI), Root Mean Square Error of Approximation (RMSEA) and Standardized Root Mean Square Residual (SRMR). According to Hu and Bentler [42], when χ^2^/df < 3, GFI > 0.90, AGFI > 0.90, CFI > 0.90, RMSEA< 0.08, and SRMR < 0.08, the model was of acceptable fit. Mediation analysis was conducted using the PROCESS Procedure for SPSS (2.16.2). All statistical analyses were performed with the significance level set at *p* < 0.05 (two-tailed).

## Results

### Correlations among psychological variables at three time points

Table 1 shows the means, standard deviations and Pearson correlation coefficients of academic burnout, resilience and life satisfaction among medical students at three measurement time points. As can be seen from the table, all psychological variables were significantly correlated with each other (*p* < 0.001) and in the expected directions. Specifically, academic burnout was significantly negatively associated with both resilience (r range: −.631 ~ −.451) and life satisfaction (r range: −.542 ~ −.350), while resilience was significantly positively related to life satisfaction (r range: .393 ~ .662) in medical students.

**Table 1:** Means (M), standard deviations (SD) and correlations of psychological variables at three time points

Variables	1.	2.	3.	4.	5.	6.	7.	8.	9.
1. AB (T1)	**1**								
2. AB (T2)	.613^***^	**1**							
3. AB (T3)	.703^***^	.680^***^	**1**						
4. RE (T1)	−.562^***^	−.468^***^	−.464^***^	**1**					
5. RE (T2)	−.480^***^	−.631^***^	−.553^***^	.620^***^	**1**				
6. RE (T3)	−.468^***^	−.451^***^	−.535^***^	.537^***^	.576^***^	**1**			
7. LS (T1)	−.514^***^	−.350^***^	−.409^***^	.560^***^	.410^***^	.406^***^	**1**		
8. LS (T2)	−.384^***^	−.542^***^	−.449^***^	.427^***^	.595^***^	.393^***^	.494^***^	**1**	
9. LS (T3)	−.422^***^	−.440^***^	−.494^***^	.404^***^	.454^***^	.662^***^	.514^***^	.509^***^	**1**
M	**49.72**	**48.96**	**48.47**	**89.09**	**88.25**	**90.42**	**22.72**	**23.34**	**24.32**
SD	**9.81**	**10.48**	**10.96**	**14.69**	**13.19**	**15.79**	**6.03**	**5.79**	**6.03**

Note. *AB* academic burnout, *RE* resilience, *LS* life satisfaction, *T*_*1*_ time point 1, *T*_*2*_ time point 2, *T*_*3*_ time point 3

*N* = 190

^***^p < 0.001 (two-tailed)

### Temporal associations between resilience and academic burnout

**Fig. 1:**
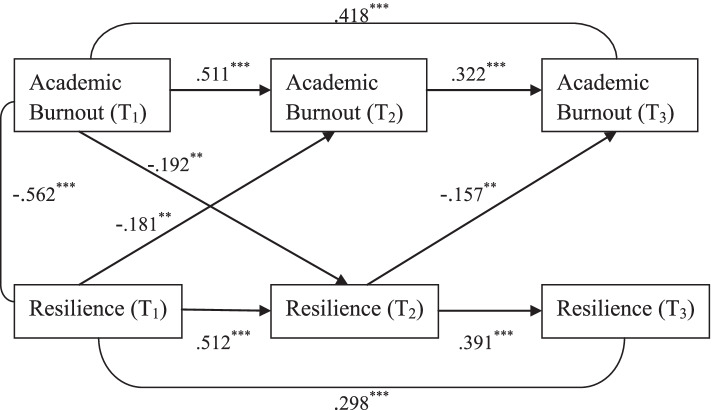
Three-wave cross-lagged model examining the temporal relationship between resilience and academic burnout in medical students. Path coefficients are standardized; ^**^p < 0.01 (two-tailed); ^***^p < 0.001 (two-tailed)

Figure 1 presents the three-wave cross-lagged model examining the temporal relationship between resilience and academic burnout among medical students. All the path coefficients in the model were standardized and statistically significant. The model demonstrated good fit, with χ^2^ /df = 2.214, *p* = 0.084, GFI = 0.989, AGFI = 0.920, CFI = 0.994, RMSEA = 0.080, SRMR = 0.032. As can be seen from the figure, the temporal relationship between academic burnout and resilience among medical students seemed reciprocal, with Academic Burnout (T_1_) negatively predicting Resilience (T_2_) (β = −.192, *p* < 0.01) and Resilience (T_1_) negatively predicting Academic Burnout (T_2_) (β = −.181, *p* < 0.01). However, between time point 2 and time point 3, while Resilience (T_2_) still negatively predicted Academic Burnout (T_3_) (β = −.157, *p* < 0.01), the path coefficient from Academic Burnout (T_2_) to Resilience (T_3_) was non-significant.

### Temporal associations between academic burnout and life satisfaction

**Fig. 2:**
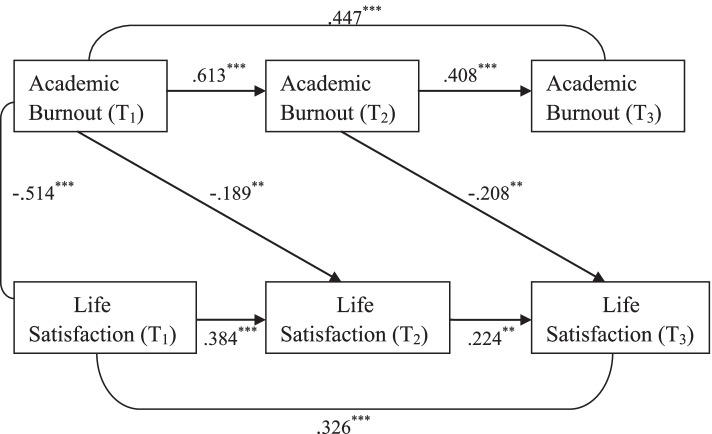
Three-wave cross-lagged model exploring the temporal relationship between academic burnout and life satisfaction in medical students. Path coefficients are standardized; ^**^p < 0.01 (two-tailed); ^***^p < 0.001 (two-tailed)

The three-wave cross-lagged model exploring the temporal relationship between academic burnout and life satisfaction among medical students was shown in Fig. 2. All the path coefficients in the model were standardized and statistically significant. The model was somewhat saturated and showed good fit, with χ^2^ /df = 0.981, *p* = 0.416, GFI = 0.993, AGFI = 0.964, CFI = 1.000, RMSEA = 0.000, SRMR = 0.024. It is clear to see from the figure that academic burnout significantly negatively predicted life satisfaction in medical students, but not the other way around. The standardized path coefficient from Academic Burnout (T_1_) to Life Satisfaction (T_2_) was significant (β = −.189, *p* < 0.01) and the standardized path coefficient from Academic Burnout (T_2_) to Life Satisfaction (T_3_) was also significant (β = −.208, *p* < 0.01), whereas the path coefficients from Life Satisfaction (T_1_) to Academic Burnout (T_2_) and from Life Satisfaction (T_2_) to Academic Burnout (T_3_) were non-significant.

### Temporal associations between resilience and life satisfaction

**Fig. 3:**
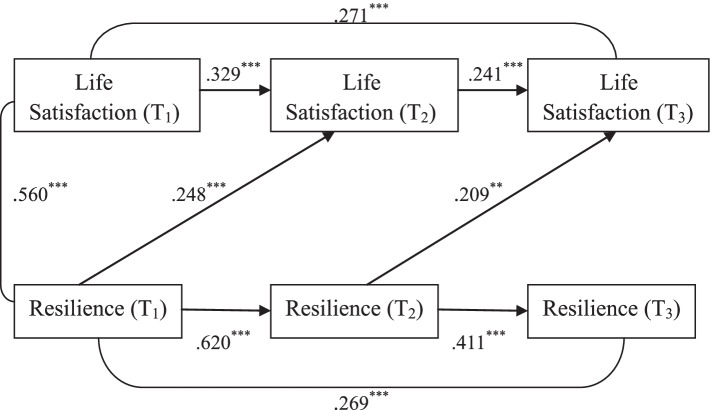
Three-wave cross-lagged model depicting the temporal relationship between life satisfaction and resilience in medical students. Path coefficients are standardized; ^**^p < 0.01 (two-tailed); ^***^p < 0.001 (two-tailed)

Figure 3 shows the three-wave cross-lagged model depicting the temporal relationship between resilience and life satisfaction among medical students. All the path coefficients in the model were standardized and statistically significant. The model demonstrated good fit, with χ^2^ /df = 1.315, *p* = 0.262, GFI = 0.991, AGFI = 0.953, CFI = 0.997, RMSEA = 0.041, SRMR = 0.034. As shown in the figure, resilience significantly positively predicted life satisfaction in medical students, but not the other way around. The standardized path coefficient from Resilience (T_1_) to Life Satisfaction (T_2_) was significant (β = .248, *p* < 0.001) and the standardized path coefficient from Resilience (T_2_) to Life Satisfaction (T_3_) was also significant (β = .209, *p* < 0.01), while the path coefficients from Life Satisfaction (T_1_) to Resilience (T_2_) and from Life Satisfaction (T_2_) to Resilience (T_3_) were non-significant.

### The mediating role of resilience between academic burnout and life satisfaction

**Fig. 4:**
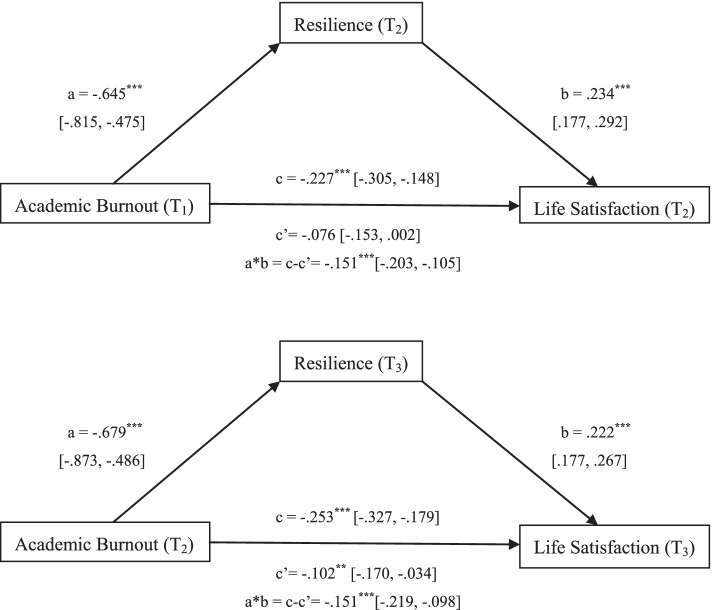
Longitudinal mediation models exploring the mediating role of resilience in the relationship between academic burnout and life satisfaction among medical students. Path coefficients are unstandardized regression coefficients with 95% confidence interval; ^**^p < 0.01 (two-tailed); ^***^p < 0.001 (two-tailed)

In order to explore the mediating role of resilience in the relationship between academic burnout and life satisfaction among medical students, two longitudinal mediation models were constructed and tested with 5000 bootstrap samples by using Model 4 in the PROCESS Procedure for SPSS (2014). Figure 4 presents the results of the mediation analyses. As can be seen from the figure, in the first longitudinal mediation model between time point 1 and time point 2, the effect of Academic Burnout at T_1_ on Resilience at T_2_ was significant (a = −.645, *p* < 0.001, 95%CI: −.815, −.475); the effect of Resilience at T_2_ on Life Satisfaction at T_2_ was significant (b = .234, *p* < 0.001, 95%CI: .177, .292); the total effect of Academic Burnout at T_1_ on Life Satisfaction at T_2_ was significant (c = −.227, *p* < 0.001, 95%CI: −.305, −.148). According to Hayes [43], the prerequisite for testifying the mediation model was met, i.e. the independent variable (academic burnout) was significantly associated with the mediating variable (resilience); the mediating variable (resilience) was significantly associated with the dependent variable (life satisfaction); the independent variable (academic burnout) was significantly associated with the dependent variable (life satisfaction). Since the direct effect of Academic Burnout at T_1_ on Life Satisfaction at T_2_ became non-significant after adding the mediator of Resilience (c’ = −.076, *p* > 0.05, 95%CI: −.153, .002) and the indirect effect of Resilience at T_2_ was significant (a*b = c-c’ = −.151, *p* < 0.001) with 95% confidence interval excluding 0 (95%CI: −.203, −.105), resilience played a full mediating role in the association of academic burnout with life satisfaction among medical students between T_1_ and T_2_ [44].

In the second longitudinal mediation model between time point 2 and time point 3, the effect of Academic Burnout at T_2_ on Resilience at T_3_ was significant (a = −.679, *p* < 0.001, 95%CI: −.873, −.486); the effect of Resilience at T_3_ on Life Satisfaction at T_3_ was significant (b = .222, *p* < 0.001, 95%CI: .177, .267); the total effect of Academic Burnout at T_2_ on Life Satisfaction at T_3_ was significant (c = −.253, *p* < 0.001, 95%CI: −.327, −.179), so the prerequisite for testifying the mediation model was met [43]. Since the direct effect of Academic Burnout at T_2_ on Life Satisfaction at T_3_ was still significant after adding the mediator of Resilience (c’ = −.102, *p* < 0.01, 95%CI: −.170, −.034) and the indirect effect of Resilience at T_3_ was significant (a*b = c-c’ = −.151, *p* < 0.001) with 95% confidence interval excluding 0 (95%CI: −.219, −.098), resilience played a partial mediating role in the association of academic burnout with life satisfaction among medical students between T_2_ and T_3_ [44].

### Supplementary analyses

We have also tested the differences in academic burnout, resilience and life satisfaction regarding gender for each of the three waves, but results show that there were no significant differences in these three psychological variables in terms of gender for the three waves (for details, please refer to the Additional file 2). As for the changes in the levels of academic burnout, resilience and life satisfaction regarding gender over the three waves, we found that the levels of these three psychological variables showed stability for both male and female medical students at the three measurement points over a span of 20-month period. However, we detected a small difference in the level of life satisfaction for medical students between time point 1 and time point 3 (for details, please refer to the Additional file 3).

## Discussion

Our study adopted a longitudinal design and used three-wave cross-lagged models to explore temporal relationships between academic burnout, resilience and life satisfaction, and to explore the possible mediating effect of resilience on the association between academic burnout and life satisfaction among medical students. To the best of our knowledge, the present study is the first one to examine the relationship among academic burnout, resilience and life satisfaction in medical students by using a three-wave longitudinal design. The results shed light on understanding the associations between a common mental health problem (academic burnout), an essential psychological resource (resilience) and a key subjective well-being indicator (life satisfaction) among medical students.

### Temporal associations between resilience and academic burnout

The result of the first three-wave cross-lagged model shows that the temporal relationship between resilience and academic burnout among medical students was somewhat reciprocal. In our study, between Time 1 and Time 2, academic burnout was found to negatively predict resilience and resilience was also found to negatively predict academic burnout. However, between Time 2 and Time 3, resilience was found to negatively predict academic burnout, but not the other way around. The possible reason for this phenomenon is that we conducted our survey at Time 3 during the COVID-19 online teaching period. Literature shows that medical students were facing great challenges when trying to adapt to the new mode of online teaching [45, 46]. The change in the relationship between resilience and academic burnout from bidirectional (Time 1 → Time 2) to unidirectional (Time 2 → Time 3) indicated that during a special period like the COVID-19 pandemic, resilience, as an important emotional regulation resource, was essential for medical students to cope with difficulties and challenges in online learning effectively, which could in turn protect them from academic burnout. Of note, here we could not make comparisons with results of other studies since we did not find any similar studies exploring the temporal relationship between resilience and academic burnout.

### Temporal associations between academic burnout and life satisfaction

The result of the second three-wave cross-lagged model shows that the temporal relationship between academic burnout and life satisfaction was unidirectional, i.e. academic burnout was found to be a negative predictor of life satisfaction among medical students, but not the other way around. This finding was in line with the conclusion of the seven-year longitudinal study conducted by Hakanen and Schaufeli [47], which found that burnout was a significant negative predictor of life satisfaction among Finnish dentists. Although some cross-sectional studies have also demonstrated the negative correlation of burnout with life satisfaction among medical students and among nurses [26, 27, 48], to the best of our knowledge, there has been no research examining the temporal relationship between academic burnout and life satisfaction among medical students by using a three-wave cross-lagged model. Our finding that academic burnout longitudinally and negatively predicted life satisfaction in medical students suggested that medical institutions and related authorities should pay attention to academic burnout problems among medical students which could result in lower life satisfaction levels.

### Temporal associations between resilience and life satisfaction

The third three-wave cross-lagged model reveals that the temporal relationship between resilience and life satisfaction was unidirectional, i.e. resilience was found to be a positive predictor of life satisfaction among medical students, but not the other way around. This finding was consistent with the result of a six-year longitudinal study by Shek and Liang [49], which found that resilience was a significant positive predictor of life satisfaction, but the sample in this study was Chinese adolescents. Another two-wave cross-lagged study by Ríos-Risquez et al. [24] concluded that resilience had a significant positive effect on psychological well-being among nursing students. However, we did not find any longitudinal studies exploring the temporal relationship between resilience and life satisfaction among medical students. Our finding that resilience could uni-directionally and positively predicted life satisfaction among medical students suggested that medical educators may consider implementing resilience-enhancing programs to raise medical students’ resilience levels in order to improve their subjective well-being.

### The mediating role of resilience between academic burnout and life satisfaction

Our study shows that resilience, as an important psychological resource, played a significant mediating role in the relationship between academic burnout and life satisfaction among medical students. Lower levels of academic burnout were associated with higher levels of resilience, which in turn contributed to higher life satisfaction levels and higher levels of academic burnout were associated with lower levels of resilience, which in turn led to lower life satisfaction levels. Resilience is a personal quality essential for one to cope with adversity and an individual with a high level of resilience can adapt to and overcome adversity despite under continuous stress [19, 50]. Medical students with high levels of resilience could adjust themselves when facing adversity, assess the situation calmly, adapt to the unfavorable circumstance, try their best to overcome difficulties, and persevere in reaching their goals. Thus, they were protected from negative emotions evoked by obstacles in study, which helped buffer the negative impacts of academic burnout on their life satisfaction levels. This may explain why resilience had a mediating effect on the relationship between academic burnout and life satisfaction among medical students.

### Implications of the present study for medical education

The findings of our study have important implications for medical education. First, academic burnout was found to negatively predict life satisfaction in medical students. This finding indicates that medical institutions and educators need to identify effective measures to reduce medical students’ academic burnout levels in order to enhance their life satisfaction levels. Literature shows that academic burnout can be caused by both external factors such as heavy academic workload and a stressful learning environment [51], and internal factors such as perceived incapability to deal with learning pressure effectively and the adoption of inappropriate coping styles [14]. To address the external factors, it is suggested that medical educators carry out the medical curricular reform to reduce academic workload and create a good and harmonious learning environment for medical students. To address the internal factors, intervention programs need to be developed to help cultivate medical students’ psychological resources and promote the use of adaptive coping strategies. Second, our study found that resilience could positively predict life satisfaction and although the temporal relationship between resilience and academic burnout was somewhat bidirectional, resilience was found to longitudinally mediate the association between academic burnout and life satisfaction among medical students. These findings suggest that we may consider implementing interventions to raise medical students’ resilience in order to buffer the negative effect of academic burnout and improve their life satisfaction levels. According to the Transactional Model Theory, under stressful circumstances, personal characteristics and psychological resources affect how an individual perceived stress, appraised the circumstance, how to react and with what kind of coping strategies [52, 53]. Interventional studies show that promoting resilience, an important psychological resource, can be effective in helping medical students alleviate psychological distress [54] and improve subjective well-being [55]. Therefore, medical institutions may consider providing resilience-promotion programs such as mindfulness-based intervention and emotional training to nurture medical students’ resilience in order to help them relieve stress, reduce burnout and enhance life satisfaction. Third, the time span for the present longitudinal study was 20 months, covering the whole preclinical education period, and the results of our supplementary analyses show that over this time period, medical students’ levels of academic burnout, resilience and life satisfaction were rather stable. For medical students, preclinical education period, i.e. the first two academic years of study, is a crucial preparation stage. Some research shows that when medical students step into the third academic year, the critical transitional stage from preclinical to clinical study, there was a downward trend in mental health indicator such as life satisfaction [26]. Therefore, it is recommended that medical institutions implement interventions early in the preclinical education period to better prepare medical students for the challenging transitional stage from preclinical to clinical study. Empirical studies show that interventions like providing classroom-based positive psychology courses and mental health education courses are effective in helping preclinical medical students reduce academic burnout levels and enhance life satisfaction levels [41, 56]. Medical educators and researchers need to make continuous efforts to experiment with different interventions and identify effective ones to implement in medical education.

### Limitations and future directions

First, self-reported questionnaires were distributed as our data collection method, so response bias and social desirability bias cannot be avoided. Second, the sample of medical students in the current study came from only one medical university in China, so generalizations about conclusions need to be made with caution. Third, in our study, the temporal associations of only one personal psychological resource of resilience with academic burnout and life satisfaction among medical students were explored, but previous studies show that psychological resources such as self-efficacy and self-esteem are also correlated with academic burnout and life satisfaction, which can be examined in future studies. Fourth, the present longitudinal study only covers the preclinical education over a period of 20 months, longitudinal research spanning a longer period of time covering both preclinical education and clinical education is recommended.

## Conclusions

This study adopted a three-wave longitudinal design to examine the temporal relationships between academic burnout, resilience and life satisfaction, and to explore the possible mediating role of resilience in the association of academic burnout with life satisfaction among medical students. Results show that academic burnout uni-directionally and negatively predicted life satisfaction, while resilience uni-directionally and positively predicted life satisfaction. However, the temporal relationship between academic burnout and resilience among medical students was somewhat bidirectional. Resilience was found to have a significant mediating effect on the relationship between academic burnout and life satisfaction. It is suggested that medical educators pay attention to medical students’ academic burnout problems and they may consider implementing resilience-promotion interventions in medical education to buffer the negative effect of academic burnout and improve medical students’ life satisfaction levels.

## Supplementary Information

**Additional file 1.** Results of the homogeneity tests of 190 medical students as the representative for 212, 222 and 216 subjects respectively in the three waves.

**Additional file 2.** Differences in medical students' academic burnout, resilience and life satisfaction regarding gender for each wave.

**Additional file 3.** Changes in medical students' academic burnout, resilience and life satisfaction regarding gender over the three waves.

## Data Availability

The datasets used and/or analyzed in the present study are available from the corresponding author on reasonable request.

## Abbreviations

CCSABI: Chinese College Student Academic Burnout Inventory
CD-RISC: Connor-Davidson Resilience Scale
SWLS: Satisfaction With Life Scale
MBI-SS: Maslach Burnout Inventory Student Survey
GFI: Goodness of Fit Index
AGFI: Adjusted Goodness of Fit Index
CFI: Comparative Fit Index
RMSEA: Root Mean Square Error of Approximation
SRMR: Standardized Root Mean Square Residual
