# Supplementary material for: Associations between academic burnout, resilience and life satisfaction among medical students: a three-wave longitudinal study
Source: BMC Med Educ. 2022 Apr 5;22:248. doi: 10.1186/s12909-022-03326-6 (PMC8980514; doi:10.1186/s12909-022-03326-6)
Supplement: Supplementary file 1 — Additional file 1. Results of the homogeneity tests of 190 medical students as the representative for 212, 222 and 216 subjects respectively in the three waves. [file 12909_2022_3326_MOESM1_ESM.doc]

**Results of independent samples t-tests for Wave1**

| **Group Statistics** | | | | | |
| --- | --- | --- | --- | --- | --- |
|  | Group | N | Mean | Std. Deviation | Std. Error Mean |
| AB(T1) | 1 | 212 | 49.41 | 9.859 | .677 |
|  | 2 | 190 | 49.72 | 9.812 | .712 |

| **Independent Samples Test** | | | | | | | | | | |
| --- | --- | --- | --- | --- | --- | --- | --- | --- | --- | --- |
|  | | Levene's Test for Equality of Variances | | t-test for Equality of Means | | | | | | |
|  |  | F | Sig. | t | df | Sig. (2-tailed) | Mean Difference | Std. Error Difference | 95% Confidence Interval of the Difference | |
|  |  |  |  |  |  |  |  |  | Lower | Upper |
| AB(T1) | Equal variances assumed | .006 | .941 | -.321 | 400 | .748 | -.315 | .983 | -2.247 | 1.617 |
|  | Equal variances not assumed |  |  | -.321 | 395.632 | .748 | -.315 | .982 | -2.247 | 1.616 |

Note: AB: academic burnout; T1: time point 1.

| **Group Statistics** | | | | | |
| --- | --- | --- | --- | --- | --- |
|  | Group | N | Mean | Std. Deviation | Std. Error Mean |
| R(T1) | 1 | 212 | 89.45 | 14.991 | 1.030 |
|  | 2 | 190 | 89.09 | 14.688 | 1.066 |

| **Independent Samples Test** | | | | | | | | | | |
| --- | --- | --- | --- | --- | --- | --- | --- | --- | --- | --- |
|  | | Levene's Test for Equality of Variances | | t-test for Equality of Means | | | | | | |
|  |  | F | Sig. | t | df | Sig. (2-tailed) | Mean Difference | Std. Error Difference | 95% Confidence Interval of the Difference | |
|  |  |  |  |  |  |  |  |  | Lower | Upper |
| R(T1) | Equal variances assumed | .052 | .820 | .245 | 400 | .807 | .363 | 1.483 | -2.553 | 3.280 |
|  | Equal variances not assumed |  |  | .245 | 396.820 | .806 | .363 | 1.482 | -2.550 | 3.276 |

Note: R: resilience; T1: time point 1.

| **Group Statistics** | | | | | |
| --- | --- | --- | --- | --- | --- |
|  | Group | N | Mean | Std. Deviation | Std. Error Mean |
| LS(T1) | 1 | 212 | 22.91 | 6.041 | .415 |
|  | 2 | 190 | 22.72 | 6.026 | .437 |

| **Independent Samples Test** | | | | | | | | | | |
| --- | --- | --- | --- | --- | --- | --- | --- | --- | --- | --- |
|  | | Levene's Test for Equality of Variances | | t-test for Equality of Means | | | | | | |
|  |  | F | Sig. | t | df | Sig. (2-tailed) | Mean Difference | Std. Error Difference | 95% Confidence Interval of the Difference | |
|  |  |  |  |  |  |  |  |  | Lower | Upper |
| LS(T1) | Equal variances assumed | .000 | .985 | .315 | 400 | .753 | .190 | .603 | -.995 | 1.375 |
|  | Equal variances not assumed |  |  | .315 | 395.440 | .753 | .190 | .603 | -.995 | 1.375 |

Note: LS: life satisfaction; T1: time point 1.

**Results of independent samples t-tests for Wave2**

| **Group Statistics** | | | | | |
| --- | --- | --- | --- | --- | --- |
|  | Group： | N | Mean | Std. Deviation | Std. Error Mean |
| AB(T2) | 1 | 222 | 49.20 | 10.654 | .715 |
|  | 2 | 190 | 48.96 | 10.481 | .760 |

| **Independent Samples Test** | | | | | | | | | | |
| --- | --- | --- | --- | --- | --- | --- | --- | --- | --- | --- |
|  | | Levene's Test for Equality of Variances | | t-test for Equality of Means | | | | | | |
|  |  | F | Sig. | t | df | Sig. (2-tailed) | Mean Difference | Std. Error Difference | 95% Confidence Interval of the Difference | |
|  |  |  |  |  |  |  |  |  | Lower | Upper |
| AB(T2) | Equal variances assumed | .106 | .745 | .234 | 410 | .815 | .245 | 1.045 | -1.810 | 2.299 |
|  | Equal variances not assumed |  |  | .235 | 402.131 | .815 | .245 | 1.044 | -1.807 | 2.297 |

Note: AB: academic burnout; T2: time point 2.

| **Group Statistics** | | | | | |
| --- | --- | --- | --- | --- | --- |
|  | Group： | N | Mean | Std. Deviation | Std. Error Mean |
| R(T2) | 1 | 222 | 87.93 | 14.007 | .940 |
|  | 2 | 190 | 88.25 | 13.189 | .957 |

| **Independent Samples Test** | | | | | | | | | | |
| --- | --- | --- | --- | --- | --- | --- | --- | --- | --- | --- |
|  | | Levene's Test for Equality of Variances | | t-test for Equality of Means | | | | | | |
|  |  | F | Sig. | t | df | Sig. (2-tailed) | Mean Difference | Std. Error Difference | 95% Confidence Interval of the Difference | |
|  |  |  |  |  |  |  |  |  | Lower | Upper |
| R(T2) | Equal variances assumed | .558 | .455 | -.238 | 410 | .812 | -.320 | 1.348 | -2.969 | 2.329 |
|  | Equal variances not assumed |  |  | -.239 | 406.254 | .811 | -.320 | 1.341 | -2.957 | 2.317 |

Note: R: resilience; T2: time point 2.

| **Group Statistics** | | | | | |
| --- | --- | --- | --- | --- | --- |
|  | Group： | N | Mean | Std. Deviation | Std. Error Mean |
| LS(T2) | 1 | 222 | 23.40 | 5.770 | .387 |
|  | 2 | 190 | 23.34 | 5.792 | .420 |

| **Independent Samples Test** | | | | | | | | | | |
| --- | --- | --- | --- | --- | --- | --- | --- | --- | --- | --- |
|  | | Levene's Test for Equality of Variances | | t-test for Equality of Means | | | | | | |
|  |  | F | Sig. | t | df | Sig. (2-tailed) | Mean Difference | Std. Error Difference | 95% Confidence Interval of the Difference | |
|  |  |  |  |  |  |  |  |  | Lower | Upper |
| LS(T2) | Equal variances assumed | .002 | .961 | .112 | 410 | .911 | .064 | .571 | -1.059 | 1.187 |
|  | Equal variances not assumed |  |  | .112 | 399.764 | .911 | .064 | .571 | -1.059 | 1.187 |

Note: LS: life satisfaction; T2: time point 2.

**Results of independent samples t-tests for Wave3**

| **Group Statistics** | | | | | |
| --- | --- | --- | --- | --- | --- |
|  | Group： | N | Mean | Std. Deviation | Std. Error Mean |
| AB(T3) | 1 | 216 | 48.76 | 11.146 | .758 |
|  | 2 | 190 | 48.47 | 10.962 | .795 |

| **Independent Samples Test** | | | | | | | | | | |
| --- | --- | --- | --- | --- | --- | --- | --- | --- | --- | --- |
|  | | Levene's Test for Equality of Variances | | t-test for Equality of Means | | | | | | |
|  |  | F | Sig. | t | df | Sig. (2-tailed) | Mean Difference | Std. Error Difference | 95% Confidence Interval of the Difference | |
|  |  |  |  |  |  |  |  |  | Lower | Upper |
| AB(T3) | Equal variances assumed | .111 | .739 | .264 | 404 | .792 | .290 | 1.100 | -1.872 | 2.453 |
|  | Equal variances not assumed |  |  | .264 | 398.990 | .792 | .290 | 1.099 | -1.870 | 2.451 |

Note: AB: academic burnout; T3: time point 3

| **Group Statistics** | | | | | |
| --- | --- | --- | --- | --- | --- |
|  | Group： | N | Mean | Std. Deviation | Std. Error Mean |
| R(T3) | 1 | 216 | 90.44 | 16.010 | 1.089 |
|  | 2 | 190 | 90.42 | 15.785 | 1.145 |

| **Independent Samples Test** | | | | | | | | | | |
| --- | --- | --- | --- | --- | --- | --- | --- | --- | --- | --- |
|  | | Levene's Test for Equality of Variances | | t-test for Equality of Means | | | | | | |
|  |  | F | Sig. | t | df | Sig. (2-tailed) | Mean Difference | Std. Error Difference | 95% Confidence Interval of the Difference | |
|  |  |  |  |  |  |  |  |  | Lower | Upper |
| R(T3) | Equal variances assumed | .021 | .884 | .012 | 404 | .991 | .019 | 1.582 | -3.091 | 3.129 |
|  | Equal variances not assumed |  |  | .012 | 398.768 | .991 | .019 | 1.581 | -3.088 | 3.126 |

Note: R: resilience; T3: time point 3.

| **Group Statistics** | | | | | |
| --- | --- | --- | --- | --- | --- |
|  | Group： | N | Mean | Std. Deviation | Std. Error Mean |
| LS(T3) | 1 | 216 | 24.47 | 5.990 | .408 |
|  | 2 | 190 | 24.32 | 6.031 | .438 |

| **Independent Samples Test** | | | | | | | | | | |
| --- | --- | --- | --- | --- | --- | --- | --- | --- | --- | --- |
|  | | Levene's Test for Equality of Variances | | t-test for Equality of Means | | | | | | |
|  |  | F | Sig. | t | df | Sig. (2-tailed) | Mean Difference | Std. Error Difference | 95% Confidence Interval of the Difference | |
|  |  |  |  |  |  |  |  |  | Lower | Upper |
| LS(T3) | Equal variances assumed | .001 | .977 | .254 | 404 | .800 | .152 | .598 | -1.023 | 1.327 |
|  | Equal variances not assumed |  |  | .254 | 396.712 | .800 | .152 | .598 | -1.024 | 1.327 |

Note: LS: life satisfaction; T3: time point 3.
