# Supplementary material for: Associations between academic burnout, resilience and life satisfaction among medical students: a three-wave longitudinal study
Source: BMC Med Educ. 2022 Apr 5;22:248. doi: 10.1186/s12909-022-03326-6 (PMC8980514; doi:10.1186/s12909-022-03326-6)
Supplement: Supplementary file 2 — Additional file 2. Differences in medical students' academic burnout, resilience and life satisfaction regarding gender for each wave. [file 12909_2022_3326_MOESM2_ESM.doc]

Table1 Gender differences in academic burnout, resilience and life satisfaction for Wave 1

|  | N (%) | AB (M ± SD) | RE (M ± SD) | LS (M ± SD) |
| --- | --- | --- | --- | --- |
| Gender |  |  |  |  |
| Male | 88(41.5) | 48.42 ± 10.29 | 89.09 ± 13.21 | 23.31 ± 6.07 |
| Female | 124(58.5) | 50.10 ± 9.53 | 89.71 ± 16.18 | 22.62 ± 6.03 |
| t |  | -1.227 | -0.295 | 0.814 |
| p |  | 0.221 | 0.768 | 0.417 |

Note. M ± SD: mean ± standard deviation; AB: academic burnout; RE: resilience; LS: life satisfaction

Table 2 Gender differences in academic burnout, resilience and life satisfaction for Wave 2

|  | N (%) | AB (M ± SD) | RE (M ± SD) | LS (M ± SD) |
| --- | --- | --- | --- | --- |
| Gender |  |  |  |  |
| Male | 94(42.3) | 50.30 ± 10.43 | 86.04 ± 13.21 | 23.28 ± 6.10 |
| Female | 128(57.7) | 48.40 ± 10.79 | 89.32 ± 14.45 | 23.49 ± 5.54 |
| t |  | 1.315 | -1.731 | -0.275 |
| p |  | 0.190 | 0.085 | 0.784 |

Note. M ± SD: mean ± standard deviation; AB: academic burnout; RE: resilience; LS: life satisfaction

Table 3 Gender differences in academic burnout, resilience and life satisfaction for Wave 3

|  | N (%) | AB (M ± SD) | RE (M ± SD) | LS (M ± SD) |
| --- | --- | --- | --- | --- |
| Gender |  |  |  |  |
| Male | 94(43.5) | 48.51 ± 10.58 | 88.18 ± 15.76 | 24.29 ± 6.15 |
| Female | 122(56.5) | 48.96 ± 11.61 | 92.18 ± 16.05 | 24.61 ± 5.88 |
| t |  | -0.293 | -1.830 | -0.388 |
| p |  | 0.770 | 0.069 | 0.699 |

Note. M ± SD: mean ± standard deviation; AB: academic burnout; RE: resilience; LS: life satisfaction
