# Supplementary material for: Associations between academic burnout, resilience and life satisfaction among medical students: a three-wave longitudinal study
Source: BMC Med Educ. 2022 Apr 5;22:248. doi: 10.1186/s12909-022-03326-6 (PMC8980514; doi:10.1186/s12909-022-03326-6)
Supplement: Supplementary file 3 — Additional file 3. Changes in medical students' academic burnout, resilience and life satisfaction regarding gender over the three waves. [file 12909_2022_3326_MOESM3_ESM.doc]

Table1 Comparisons of change in academic burnout levels regarding gender in three waves

|  | AB (M ± SD)  T1 | AB (M ± SD)  T2 | AB (M ± SD)  T3 | F | p |
| --- | --- | --- | --- | --- | --- |
| Male | 49.10 ± 10.00 | 49.40 ± 10.61 | 47.67 ± 10.40 | 0.647 | 0.524 |
| Female | 50.18 ± 9.69 | 48.63 ± 10.42 | 49.07 ± 11.38 | 0.629 | 0.534 |
| Male & Female | 49.72 ± 9.81 | 48.96 ± 10.48 | 48.47 ± 10.96 | 0.691 | 0.502 |
| Note. Male: n = 81; Female: n = 109; M ±SD: mean ± standard deviation; AB: academic burnout; T1: time point 1;  T2: time point 2; T3: time point 3 | | | | | |

Table2 Comparisons of change in resilience levels regarding gender in three waves

|  | RE (M ± SD)  T1 | RE (M ± SD)  T2 | RE (M ± SD)  T3 | F | p |
| --- | --- | --- | --- | --- | --- |
| Male | 89.02 ± 12.27 | 87.23 ± 11.84 | 88.36 ± 15.23 | 0.380 | 0.684 |
| Female | 89.14 ± 16.31 | 89.01 ± 14.12 | 91.95 ± 16.08 | 1.252 | 0.287 |
| Male & Female | 89.09 ± 14.69 | 88.25 ± 13.19 | 90.42 ± 15.79 | 1.067 | 0.345 |

Note. Male: n = 81; Female: n = 109; M ±SD: mean ± standard deviation; RE: resilience; T1: time point 1; T2: time point 2; T3: time point 3

Table3 Comparisons of change in life satisfaction levels regarding gender in three waves

|  | LS (M ± SD)  T1 | LS (M ± SD)  T2 | | LS (M ± SD)  T3 | | F | p |
| --- | --- | --- | --- | --- | --- | --- | --- |
| Male | 22.91 ± 6.00 | 23.42 ± 6.13 | | 24.28 ± 6.16 | | 1.046 | 0.353 |
| Female | 22.57 ± 6.07 | 23.28 ± 5.56 | | 24.34 ± 5.96 | | 2.516 | 0.082 |
| Male & Female | 22.72 ± 6.03 | 23.34 ± 5.79 | | 24.32 ± 6.03 | | 3.491 | 0.031 |
|  | Post-hoc Bonferroni test | | | | | | |
|  | LS (T1 vs T2) | | LS (T1 vs T3) | | LS (T2 vs T3) | | |
| Male & Female | Mean Difference: -0.621  p: 0.928 | | Mean Difference: -1.600  p: 0.027 | | Mean Difference: -0.979  p: 0.328 | | |

Note. Male: n = 81; Female: n = 109; M ±SD: mean ± standard deviation; LS: life satisfaction; T1: time point 1; T2: time point 2; T3: time point 3
